# Examining the impact of loneliness and resilience on mental health: Empirical evidence from a nationally representative sample of American college students

**DOI:** 10.1192/j.eurpsy.2025.878

**Published:** 2025-08-26

**Authors:** P. J. Sullivan, J. Ruiz-Menjivar, X. Zhang, B. R. Carr, M. Abraczinskas

**Affiliations:** 1 Department of Family, Youth and Community Sciences; 2 Department of Psychiatry / Anesthesiology, University of Florida, Gainesville, United States

## Abstract

**Introduction:**

Mental health disorders, including anxiety and major depressive disorder, are highly prevalent among college students, often leading to significant impairments in academic functioning and psychosocial well-being. Loneliness, characterized as subjective distress arising from a perceived deficit in social connectivity, is frequently associated with the exacerbation of psychiatric symptoms. In contrast, psychological resilience, defined as the capacity to adaptively manage stress and adversity, is increasingly recognized as a key protective factor against the development of psychopathology.

**Objectives:**

Despite understanding the roles of loneliness and resilience, their combined effects on mental health, specifically anxiety and depression, have not been fully explored in a large-scale, diverse population of college students in the United States. This study seeks to address this gap.

**Methods:**

Using data from the 2023-2024 Healthy Mind Study (N=104,729), we employed logistic regression to assess the predictors of anxiety and depression, focusing on two key predictors: loneliness and resilience. Our models also controlled for other relevant factors, such as campus climate, financial stress, and sociodemographic control variables, including sex, race/ethnicity, and traditional student status. Analysis was conducted with a sample delimited to undergraduate students (n=22,927).

**Results:**

Feeling lonely was positively related to moderate-to-severe depression (β = 2, p < 0.001) and moderate-to-severe anxiety (β = 1.45, p < 0.001). Resilience was a protective factor and was negatively associated with self-reported moderate-to-severe depression (β = -1.54, p < 0.001) and moderate-to-severe anxiety (β = -1.54, p < 0.001). The effect of loneliness and resilience on depression and anxiety remains consistent with the baseline models after controlling for campus climate, financial stress, and sociodemographic variables. High levels of financial stress and perceived poor campus climate were positively related to moderate-to-severe depression and anxiety. Finally, female, non-White, and non-traditional-aged students were less likely to exhibit moderate-to-severe depression and anxiety.

**Conclusions:**

The findings highlight the importance of loneliness and resilience in shaping mental health outcomes among undergraduate college students. Loneliness was negatively associated with the evaluated mental health burdens, while resilience emerged as a protective factor against these outcomes. Our findings underscore the importance of considering loneliness, resilience, financial stress, and campus climate as variables of interest when designing mental health interventions to improve academic performance and overall well-being among undergraduate college students.

**Disclosure of Interest:**

None Declared

